# A Practical Treatise on Operative Dentistry

**Published:** 1868-05

**Authors:** J. Taft

**Affiliations:** Professor of Operative Dentistry and Dental Hygiene in the Ohio College of Dental Surgery


### A PRACTICAL TREATISE ON OPERATIVE DENTISTRY.

BY J. TAFT,

Professor of Operative Dentistry and Dental Hygiene in the Ohio College of Dental Surgery.

Second Edition, with eighty-six illustrations. Philadelphia: LINDSAY & BLAKISTON. 1868.

WHEN the first edition of this work appeared, nine years ago, it imparted a new impulse to the practice of Operative Dentistry. In the early days of the profession, excellence, as an operator, was attainable only by long years of industrious practice. Now, many of the best operators are found among those comparatively young. This is as it should be; and perhaps no single agency has done more to produce this result than this volume of Professor Taft, in systematically and clearly setting forth the attained truths of our science in such a way as to be accessible to all.

A new edition has been needed for some time--partly because the first was exhausted; but mainly, as stated in the author's preface, because "so great have been the changes in many points of practice and application of principles, that those given as the best, nine years ago, are superceded by others and out of use." The second edition contains nearly fifty additional pages of matter, and the whole work is carefully revised, with a labor, but little, if any, short of the preparation of an entirely new volume.

The publishers have done all that their high reputation promised in bringing out the book in handsome style. Type, paper, binding—everything is worthy of Lindsay & Blakiston.

Price $4.50. For sale at the Dental Depots, and by booksellers generally. W.

